# The polyphenol-rich extract from chokeberry (*Aronia melanocarpa* L*.*) modulates gut microbiota and improves lipid metabolism in diet-induced obese rats

**DOI:** 10.1186/s12986-020-00473-9

**Published:** 2020-07-07

**Authors:** Yue Zhu, Jia-ying Zhang, Yu-long Wei, Jing-yi Hao, Yu-qing Lei, Wan-bin Zhao, Yu-hang Xiao, Ai-dong Sun

**Affiliations:** College of Biological Sciences and Technology, Beijing Forestry University, Beijing, 100083 China

**Keywords:** Chokeberry, Gut microbiota, Lipid metabolism, Obese rats

## Abstract

The gut microbiota plays a critical role in obesity and lipid metabolism disorder. Chokeberry (*Aronia melanocarpa* L*.*) are rich in polyphenols with various physiological and pharmacological activities. We determined serum physiological parameters and fecal microbial components by using related kits, liquid chromatography-mass spectrometry (LC-MS) and 16S rRNA gene sequencing every 10 days. Real-time PCR analysis was used to measure gene expression of bile acids (BAs) and lipid metabolism in liver and adipose tissues. We analyzed the effects of different Chokeberry polyphenol (CBPs) treatment time on obesity and lipid metabolism in high fat diet (HFD)-fed rats. The results indicated that CBPs treatment prevents obesity, liver steatosis and improves dyslipidemia in HFD-fed rats. CBPs modulated the composition of the gut microbiota with the extended treatment time, reducing the *Firmicutes*/*Bacteroidetes* ratio (F/B ratio) and increasing the relative abundance of *Bacteroides*, *Prevotella*, *Akkermansia* and other bacterial species associated with anti-obesity properties. We found that CBPs treatment gradually decreased the total BAs pool and particularly reduced the relative content of cholic acid (CA), deoxycholic acid (DCA) and enhanced the relative content of chenodeoxycholic acid (CDCA). These changes were positively correlated *Bacteroides*, *Prevotella* and negatively correlated with *Clostridium*, *Eubacterium*, *Ruminococcaceae*. In liver and white adipose tissues, the gene expression of lipogenesis, lipolysis and BAs metabolism were regulated after CBPs treatment in HFD-fed rats, which was most likely mediated through FXR and TGR-5 signaling pathway to improve lipid metabolism. In addition, the mRNA expression of PPARγ, UCP1 and PGC-1α were upregulated markedly in interscapular brown adipose tissue (iBAT) after CBPs treatment. We confirmed that CBPs could reduce the body weight of HFD-fed rats by accelerating energy homeostasis and thermogenesis in iBAT. Finally, the fecal microbiota transplantation (FMT) experiment results demonstrated that FMT from CBPs-treated rats failed to reduce the weight of HFD-fed rats. However, FMT from CBPs-treated rats improved dyslipidemia and reshaped gut microbiota in HFD-fed rats. In conclusion, CBPs treatment improved obesity and complications by regulating gut microbiota in HFD-fed rats. The gut microbiota plays an important role in BAs metabolism after CBPs treatment, and BAs have therefore emerged as major effectors in microbe-host signaling events that influence host lipid metabolism, energy metabolism and thermogenesis.

## Introduction

Obesity, a state of chronic subclinical inflammation, is the key element associated with the development of various metabolic disorders [1, 2]. Lipid metabolism disorders is intimately present in obesity, which are accompanied by symptoms of dyslipidemia that include exceeding serum levels of total cholesterol (TC), triglyceride (TG), low density lipoprotein cholesterol (LDL-C), and lower level of high density lipoprotein cholesterol (HDL-C). These symptoms are induced by the dysregulation of hepatic lipid metabolism [3, 4]. In addition, white adipose tissue (WAT) is the vital site of lipid metabolism. Once the balance between lipogenesis and lipodieresis is broken, adipocyte hypertrophy will lead to dysfunctional endocrine signalling resulting in an increased risk of obesity and related metabolic diseases [5]. As hydroxy - methyl - glutaryl - coenzyme A (HMG-CoA) reductase inhibitors, statins are widely applied to the treatment of dyslipidemia through lowering TC and LDL-C levels [6, 7]. However, statins therapy is associated with some adverse effects including myotoxicity, diabetes mellitus, central nervous system complaints and hepatotoxicity [8, 9], which limits effectiveness in the treatment of patients with cardiovascular diseases.

In recent years, with the increasing interest in the study of gut microbiota, it has been found that gut microbiota plays an important role in human health and disease. More and more researches have indicated that gut microbiota participates in host nutrient acquisition, energy regulation, lipid metabolism and immunity [10, 11]. Dysbiosis of gut microbiota is associated with various diseases, including obesity, type 2 diabetes and inflammatory bowel disease [12–14]. High-fat diet (HFD) has become a standard model for development of obesity in rats by altering and remodeling the composition of gut microbiota [15, 16]. Obesity and associated metabolic disorders can be induced through increasing in the abundance of *Firmicutes* or the ratio of *Firmicutes* to *Bacteroidetes* (F/B ratio) in HFD-fed rats [13, 14]. Nevertheless, the exact mechanisms that link between altering in the composition of the gut microbiota and the development of obesity remain obscure as a result of the complexity of the pathologies.

Chokeberry (*Aronia melanocarpa* L.)*,* known as “superberries”, is a member of the Rosaceae family, which originates from the eastern parts of North America and East Canada [17]. Chokeberry is rich in nutritious ingredients including dietary fiber, organic acids, sugar, fat, protein, minerals and vitamins [18, 19]. Specifically, the polyphenols content of chokeberry is higher than those of other berries (blueberry, cranberry and lingonberry crops), which exhibits various physiological activities such as antioxidant, anti-inflammatory, antidiabetic, anti-cardiovascular diseases and so on [20–23]. Based on abundant phenolic substances content and various physiological effects of chokeberry, the aim of our study was to evaluate the impact of the polyphenols extract from chokeberry (CBPs) on improvement obesity and associated lipid metabolism disorders in HFD-fed rats, as well as comprehensive investigating the role of the gut microbiota in mediating the effects of the CBPs on host metabolism.

## Materials and methods

### Ethical approval

The experiments adhered to the China Institutional Animal Care Use Committee and were licensed by the Ethics Committee of Beijing Laboratory Animal Research Center (Qualified number: BLARC-2018-A033).

### Extraction of polyphenols from chokeberry and structure analysis

The polyphenols were extracted in accordance with our previous research. Briefly, frozen chokeberries (10 kg) were crushed using a beater for 3 min. Then, materials were extracted with a 13:7 (v/v) ethanol/water solution at 45 °C for 90 min (simultaneous with 30 min ultrasonic extraction). The solution was centrifuged at 4000 r/min for 20 min. The supernatant was collected, and ethanol was removed from the supernatant through rotatory evaporation under vacuum at 40 °C. The CBPs was freeze-dried and stored at − 80 °C. The structure of polyphenols in the chokeberry used in this study is described in our previous research [24]. The polyphenols profile of the chokeberry extract is available in Table 1.

**Table 1:** Chemical characterisation of the polyphenols extract from chokeberry

	Extract content (mg/100 g fresh weight)	Daily intake^**a**^ (mg/kg body weight)
Total polyphenols	2209.25	22.09
(+) - catechin	4.34	0.04
(−) - epicatechin	45.28	0.45
Chlorogenic acid	1253.17	12.53
cis-Tiliroside	13.25	0.13
Procyanidins	932.15	9.32
Procyanidin B1	9.18	0.092
Procyanidin B2	63.25	0.63
Procyanidin C1	6.07	0.06
Anthocyanin	486.21	4.86
Cyanidin-3-galactoside chloride	285.35	2.85
Cyanidin 3-monoarabinoside	90.24	0.90
Cyanidin 3-Xyloside	14.59	0.15
Cyanidin 3-O-glucoside chloride	16.57	0.17

^**a**^: Daily intake was calculated based on the 1000 mg of chokeberry polyphenols extract/kg of body weight dose orally given to mice for 40 days

### Animals and experimental design

Male wistar rats (aged 6 weeks and weighing 220 ± 20 g) were purchased from the Beijing Vital River Laboratory Animal Technology Co., Ltd. under specific pathogen-free (SPF) conditions and were housed under 12 h-light/12 h-dark cycle, 24 °C, 60% humidity. All rats were adaptively raised a week and randomly divided into two groups: (1) normal diet group (control group, *n* = 10), fed with a control diet (10% kcal from fat, 20% kcal from proteins, 70% kcal from carbohydrates). (2) high fat diet group, fed with high fat diet (45% kcal from fat, 20% kcal from proteins, 35% kcal from carbohydrates). After 2 months of continuous feeding, the obese rats model was established successfully. High fat diet group rats were randomly divided into 3 groups: (1) HF group (*n* = 8), continually fed with HFD and administered intragastrically normal saline with 2 mL/kg body weight once daily. (2) AM group (*n* = 10), continually fed with HFD and administered intragastrically CBPs with 1000 mg/kg body weight once daily. (3) SV group (n = 10), continually fed with HFD and administered intragastrically simvastatin with 5 mg/kg body weight once daily. All animals had free access to food and water. These treatment lasted for 40 days. Throughout the duration of the trial, body weight of rats were monitored weekly. Feces and blood samples were collected every 10 days. The collected fecal samples were immediately placed in liquid nitrogen and stored at − 80 °C. The blood sample were collected via posterior ophthalmic venous plexus of rats and serum was separated and stored at − 80 °C for later analysis of serum biochemical parameters. At the end of the experimental period, the liver, kidney, spleen, heart, lung, pancreas, testicle, epididymal adipose tissue (eWAT), inguinal adipose tissue (iWAT), perirenal adipose tissue (pWAT) and interscapular brown adipose tissue (iBAT) were collected after rats were killed by carbon dioxide inhalation. Viscera organizations and adipose tissues wet weight were measured using a precision balance.

### Biochemical analysis

Serum TC, TG, HDL-C, LDL-C, hepatic TC and TG were determined using the commercially available kits from Nanjing Jiancheng Bioengineering Institute (Nanjing, China). Serum bile acids were analyzed using the previously published procedure with some minor modifications [25]. The serum was melted on ice for 30–60 min. 100 μL serum was added in 300 μL methanol. Vortex for 10 min. Extracts were centrifuged at 12000 g, 4 °C for 30 min. Supernatants were then transferred sample vial for UPLC-MS analysis. A Thermo U3000 ultra performance LC system (Thermo Fisher Scientific Inc. Waltham, MA USA) was used throughout. The mass spectrometer was a Thermo Q Exactive instrument with an ESI source (Thermo Fisher Scientific Inc. Waltham, MA USA). The entire LC-MS system is controlled by Xcalibur 2.2 SP1.48 software. All chromatographic separations were performed with an ACQUITY UPLC HSS T3 C18 1.7 μm 100 × 2.1 mm (Waters Inc. Massachusetts, USA). The elution pattern was set to gradient elution and was listed in Supplementary Table 1. Chemicals and Reagents HPLC grade acetonitrile and methanol were purchased from Thermo Fisher (Thermo Fisher Scientific Inc. Waltham, MA USA). Formic acid was obtained from Sigma-Aldrich Inc.(St. Louis, MO, United States). All the bile acid standards were purchased from Steraloids Inc. (Newport, RI USA).

### Histopathological analysis

Liver and adipose tissues were fixed in 4% paraformaldehyde at room temperature for 24 h, which were dehydrated with a sequence of ethanol solutions and embedded in paraffin. Tissue sections (5–6 mm thick) were cut and stained with hematoxylin and eosin (H&E) staining. Sections were observed by a Nikon Eclipse E100 microscope (Nikon, Japan) under 400× magnification for liver and 200× magnification for adipose tissues.

### DNA extraction from fecal samples

Total genome DNA from samples was extracted from rats feces using Magen Hipure Soil DNA Kit according to manufacturer’s protocols. DNA concentration was monitored by Qubit3.0 Fluorometer.

### PCR amplification and Illumina MiSeq sequencing

20 ng DNA was used to generate amplicons. V3 and V4 hypervariable regions of prokaryotic 16S rDNA were selected for generating amplicons and following taxonomy analysis. The V3 and V4 regions were amplified using forward primers containing the sequence “CCTACGGRRBGCASCAGKVRVGAAT” and reverse primers containing the sequence “GGACTACNVGGGTWTCTAATCC”. At the same time, indexed adapters were added to the ends of the 16S rDNA amplicons to generate indexed libraries ready for downstream NGS sequencing on Illumina Miseq. PCR reactions were performed in triplicate 25 μL mixture containing 2.5 μL of TransStart Buffer, 2 μL of dNTPs, 1 μL of each primer, and 20 ng of template DNA. DNA libraries concentration were validated by Qubit3.0 Fluorometer. Quantify the library to 10 nM, DNA libraries were multiplexed and loaded on an Illumina MiSeq instrument according to manufacturer’s instructions (Illumina, San Diego, CA, USA). Sequencing was performed using PE250/300 paired-end; image analysis and base calling were conducted by the MiSeq Control Software (MCS) embedded in the MiSeq instrument.

The QIIME data analysis package was used for 16S rRNA data analysis. The forward and reverse reads were joined and assigned to samples based on barcode and truncated by cutting off the barcode and primer sequence. Quality filtering on joined sequences was performed and sequence which did not fulfill the following criteria were discarded: sequence length < 200 bp, no ambiguous bases, mean quality score ≥ 20. Then the sequences were compared with the reference database (RDP Gold database) using UCHIME algorithm to detect chimeric sequence, and then the chimeric sequences were removed.

The effective sequences were used in the final analysis. Sequences were grouped into operational taxonomic units (OTUs) using the clustering program VSEARCH (1.9.6) against the Silva 132 database pre-clustered at 97% sequence identity. The Ribosomal Database Program (RDP) classifier was used to assign taxonomic category to all OTUs at confidence threshold of 0.8. The RDP classifier uses the Silva 132 database which has taxonomic categories predicted to the species level.

Sequences were rarefied prior to calculation of alpha and beta diversity statistics. Alpha diversity indexes were calculated in QIIME from rarefied samples using for diversity the Shannon index, for richness the Chao1 index. Microbiota-based biomarker analysis was performed with LEfSe using the online analysis software: http://huttenhower.sph.harvard.edu/galaxy/root?tool_id=lefse_upload.

### Real-time PCR analysis

Total RNA was isolated from liver, eWAT, iWAT and iBAT through Trizol (SinoGene Biotech co., Ltd. China) in accordance with the manufacturer’s protocols and then treated with DNase I. The reverse transcription was implemented with the Thermo First cDNA Synthesis Kit (SinoGene Biotech co., Ltd. China). Real-time PCR was performed with StepOnePLUS Real-Time PCR System (Thermo Fisher Scientific Inc. Waltham, MA USA). β-actin gene was applied as reference. Primer sequences were listed in Supplementary Table 2.

### Fecal microbiota transplantation (FMT)

Male wistar rats (aged 6 weeks and weighing 210 ± 20 g) were randomly divided into 2 groups: (1) FMT-HF group (*n* = 8) and (2) FMT-AM group (*n* = 9), fed with a high fat diet (45% kcal from fat, 20% kcal from proteins, 35% kcal from carbohydrates). The HF and AM groups rats were considered as donor rats and their fecal samples were collected for 37–40 days after treatment with simvastatin and CBPs. Fecal samples (5 g) from donor rats were resuspended in sterile saline (25 mL) and mixed using benchtop vortex. Then, the samples were centrifugated at 3500 g and the microbiota supernatants were transplanted into the recipient rats (FMT-AM group rats and FMT-HF group rats) by clysis way every 2 days. Fresh transplant material was prepared on the same day of transplantation. Gut microbiota transplantation test lasted for 30 days. Body weight of rats were monitored and feces, blood samples were collected every 10 days. After 30 days transplantation, animals were euthanatized by carbon dioxide inhalation. Liver, kidney, spleen, eWAT, iWAT, pWAT and iBAT were collected.

### Statistics analysis

Statistical analysis was performed by using Prism version 7.0 (Graph-Pad Software, USA). One-way ANOVA were used to analyze significance to the differences by Tukey’s post hoc test for multiple comparisons. The significant differences between the groups were analyzed by two-way repeated measures ANOVA when data was measured with the change of time. *P* values of 0.05 or less were considered significant. All data are expressed as the mean ± SEM.

## Results

### CBPs prevents obesity, liver steatosis and improves dyslipidemia in HFD-fed rats

**Fig. 1:**
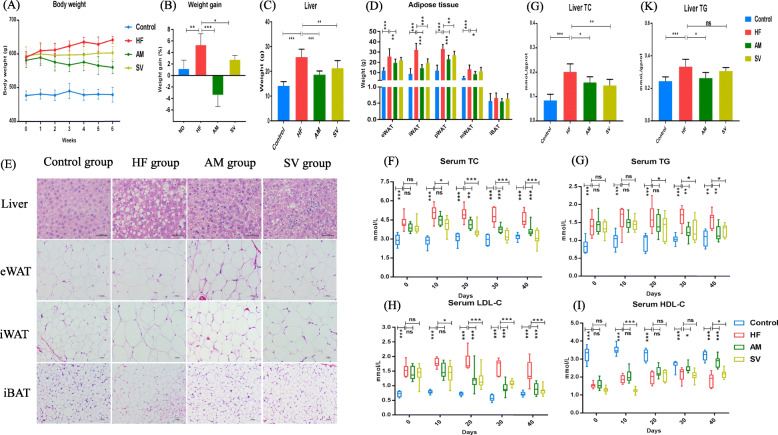
Polyphenols of *Aronia melanocarpa* treatment prevented obesity and improved hyperlipidemia in HF diet-fed rat. **a** Body weight (g), **b** Weight gain (%), **c** Liver Weight (g), **d** Adipose tissue weight (g), **e** Hepatic and adipose tissues morphology shown at × 400 or × 200 magnification, **f-i** Serum concentrations of TC, TG, HDL-C and LDL-C (mmol/L), **g-k** Hepatic concentrations TC and TG (mmol/L). Values are means ± SEMs. *P < 0.05, **p < 0.01 and ***p < 0.001

After 2 months of continuous feeding high fat diet, the body weight of rats in high fat diet group and control group were 594 ± 47.73 g and 476.72 ± 32.95 g, respectively. There was significant difference between the two groups, which indicated that the obesity model of rats was successfully established. The body weight of AM group rats decreased continuously during CBPs treatment*.* Weight gain of AM group rats has a significant difference compared with HF group rats (*P* < 0.001) (Fig. 1a, b). Notedly, the body weight of HF and SV group rats increased slowly, and weight gains were 5.29 and 2.72%, respectively, with significant difference (*P* < 0.05) (Fig. 1b). Compared with the HF group rats, the weight of visceral adipose tissues (eWAT, pWAT and mWAT), subcutaneous adipose tissue (iWAT) and liver reduced in AM group rats and SV group rats (except eWAT) (Fig. 1c, d). There were no significant differences in weight of iBAT, heart, kidney, spleen, lung, pancreas and testicle among the three groups (Fig. 1d and Supplementary Fig. 1). Overall, CBPs treatment tended to prevent weight gain by reducing the weight of liver, visceral and subcutaneous adipose tissues in HFD-fed rats. Simvastatin treatment also inhibited weight gain slightly in HFD-fed rats. However, its effect of improving obesity was inferior to CBPs treatment. There were significant difference in liver TC and TG concentration of AM and HF group rats (*p* < 0.05) (Fig. 1g, k). Similarly, H&E staining of liver and adipose tissues also showed obese rats treated with CBPs significantly reduced the hepatic fat droplets and adipocyte size compare with HF group rats (Fig. 1e). Simvastatin treatment could decreased liver TC concentration, whereas it had no effect on reducing liver TG concentration in HFD-fed rats (Fig. 1g, k). Besides, simvastatin treatment also improved the fat accumulation and reduced adipocyte size in liver and adipose tissues, which was less effective than CBPs treatment (Fig. 1e).

The serum TC, TG and LDL-C increased significantly and the serum HDL-C decreased in HF group rats compared with the control group rats (*P* < 0.001) within 40 days of HFD feeding (Fig. 1f-i), suggesting that rats fed with high-fat diet could be induced to develop hyperlipidemia. During the CBPs treatment, serum TC, TG and LDL-C decreased gradually and there were significant differences compared with HF group after 20, 30 and 20 days, respectively (Fig. 1f-h). Serum HDL-C increased gradually within 40 days of CBPs treatment, which was a significant difference between AM group and HF group after 30 days (Fig. 1i). The results manifested that CBPs treatment could improve hyperlipemia by reducing serum TC, TG, LDL-C and increasing HDL-C concentrations in HFD-fed rats. Simvastatin treatment could also significantly improve hyperlipidemia, which was more effective to reduce serum TC and LDL-C than CBPs treatment. There were significant differences in serum TC and LDL-C between SV and HF group rats after 10 days (Fig. 1f, h). However, the effect of CBPs treatment on reducing serum TG and increasing HDL-C were better than that of simvastatin treatment in HFD-fed rats (Fig. 1g, i).

### CBPs alters gut microbial composition in HFD-fed rats

We analyzed the fecal microbial composition of HF, AM, SV group rats after 10, 20, 30 and 40 days. ACE, Chao1, shannon and simpson indexes were examined for the richness and alpha-diversity of the gut microbiota. The HF group rats revealed significantly higher ACE and Chao1 indexes after 20 days. There was no significant differences of shannon and simpson diversity indexes in HF group within 40 days (Table 2). Meanwhile, the ACE, Chao1, shannon and simpson indexes were no significant differences after treatment with CBPs and simvastatin (Table 2).

**Table 2:** The ACE, Chao1, Shannon and Simpsom index in HF, AM and SV group rat at day 10, day 20, day 30, day 40 after diet intervention

Sample	HF				AM				SV			
	1HF	2HF	3HF	4HF	1 AM	2 AM	3 AM	4 AM	1SV	2SV	3SV	4SV
ACE	535.22 ± 45.03^b^	603.32 ± 23.03^a^	590.93 ± 40.91^a^	609.66 ± 25.04a	614.82 ± 27.76	611.92 ± 31.67	607.16 ± 39.07	566.10 ± 46.86	564.87 ± 51.55	595.56 ± 36.61	580.88 ± 31.95	591.88 ± 37.17
Chao1	528.70 ± 46.48^b^	591.05 ± 16.66^a^	594.38 ± 37.21^a^	623.40 ± 23.03a	614.35 ± 27.95	607.70 ± 36.86	613.21 ± 35.94	571.70 ± 48.78	570.79 ± 62.95	601.71 ± 37.46	588.21 ± 30.88	597.42 ± 35.21
Shannon index	6.02 ± 0.40	6.62 ± 0.25	6.52 ± 0.40	6.83 ± 0.24	6.81 ± 0.19	6.81 ± 0.38	6.88 ± 0.33	6.69 ± 0.44	6.47 ± 0.34	6.68 ± 0.27	6.72 ± 0.35	6.81 ± 0.36
Simpsom index	0.95 ± 0.03	0.97 ± 0.01	0.97 ± 0.01	0.98 ± 0.01	0.98 ± 0.01	0.97 ± 0.01	0.98 ± 0.01	0.98 ± 0.01	0.97 ± 0.01	0.97 ± 0.01	0.97 ± 0.01	0.98 ± 0.01

**Fig. 2:**
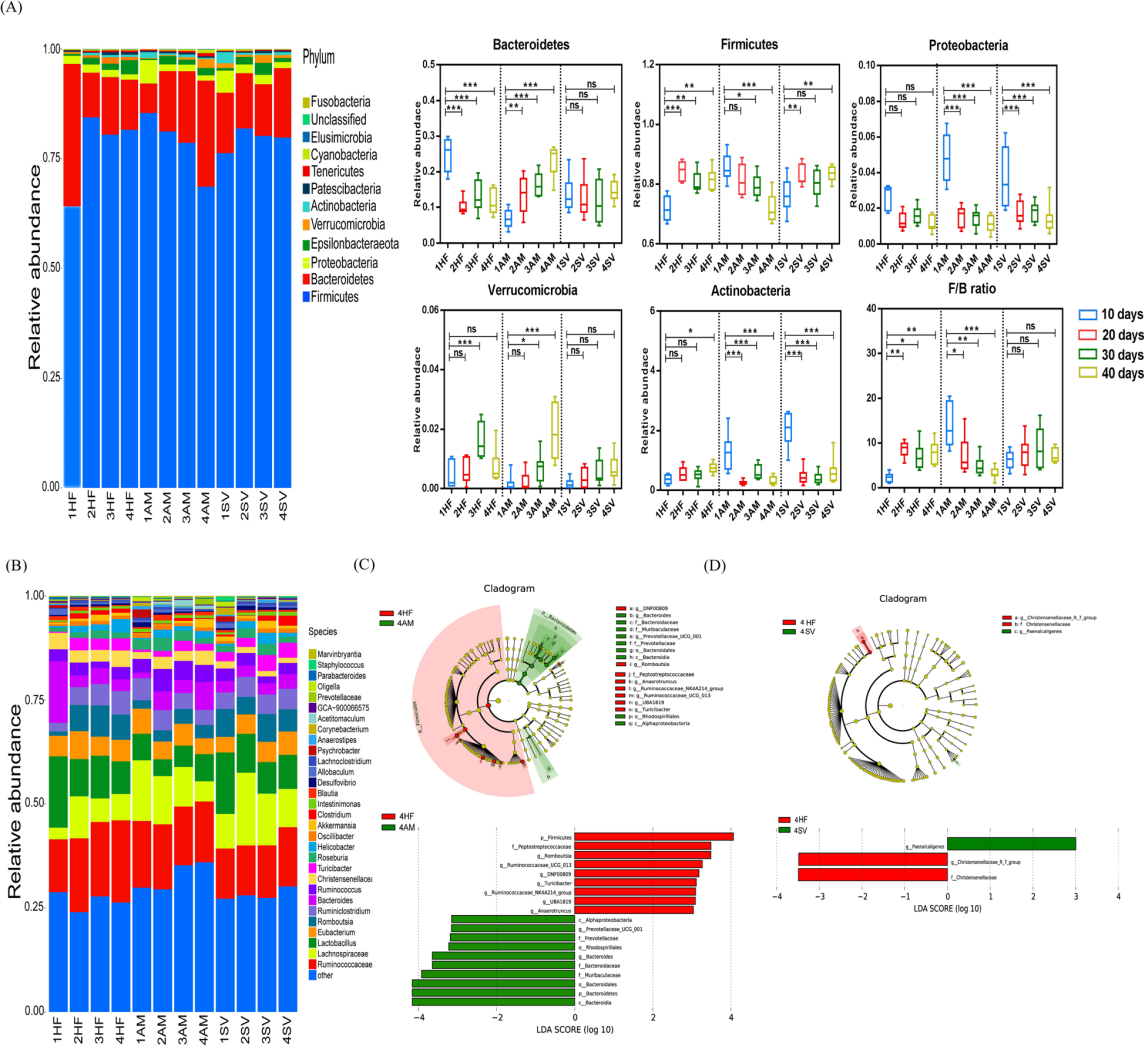
CBPs treatment improved gut microbiota in HFD-induced rat. **a** Microbiota compositions at the phylum level, **b** Microbiota compositions at the genus level in HF, AM and SV group at day 10, day 20, day 30, day 40 after diet intervention. The linear discriminant analysis (LDA) effect size (LEfSe) was used to identify the biomarkers with significant differences between the two groups: **c** 4HF vs 4 AM and **d** 4HF vs 4SV. Values are presented as mean ± SEM. *P < 0.05; **P < 0.01; ***P < 0.001; ns *P* > 0.05

CBPs supplementation had a greater effect on gut microbial composition. At the phylum level, the relative abundance of *Bacteroidetes* reduced and the relative abundance of *Firmicutes*, F/B ratio increased in HF group rats, which were no significant change after 20 days (Fig. 2a). Conversely, the relative abundance of *Bacteroidetes* and *Verrucomicrobia* were gradually increased in the AM group rats within 40 days, while the relative abundance of *Firmicutes* and F/B ratio were suppressed markedly (Fig. 2a). The relative abundance of *Proteobacteria* decreased significantly within 20 days in HF group rats, which was no significant variation after 20 days. In SV group rats, except for *Proteobacteria*, there were no significant change in relative abundance of *Bacteroidetes, Firmicutes*, *Verrucomicrobia* and F/B ratio within 40 days (Fig. 2a).

At the genus level, the gut microbial composition showed similar trends to the phylum level. The relative abundance of *Firmicutes* phylum (*Lachnospiraceae_NK4A136_group, Lachnoclostridium*), *Proteobacteria* phylum (*Desulfovibrio*) were decreased and the relative abundance of *Bacteroidetes* phylum (*Bacteroides, Prevotella*), *Firmicutes* phylum (*Romboutsia*), *Verrucomicrobia* phylum (*Akkermansia*) were increased gradually in AM group rats within 40 days (Supplementary Fig. 2). However, the relative abundance of *Firmicutes* phylum (*Lachnospiraceae_NK4A136_group, Clostridium*) were higher and *Bacteroidetes* phylum (*Bacteroides, Prevotella*), *Verrucomicrobia* phylum (*Akkermansia*) were lower in HF group rats compared with AM group rats after 40 days (Supplementary Fig. 4). Except for the increasing in the relative abundance of genus *Clostridium*, there was no significant change in other genus within 40 days of simvastatin treatment (Supplementary Fig. 2). Furthermore, the linear discriminant analysis (LDA) effect size (LEfSe) was used to identify the biomarkers with significant differences between the two groups. After 40 days, compared with the AM group rats, the relative abundance of *Firmicutes* phylum (*Romboutsia, Ruminococcaceae, Turicibacter, UBA1819, Anaerotruncus*) and *Actinobacteria* phylum (*DNF00809*) were altered significantly in HF group rats. However, the the relative abundance of *Bacteroidetes* phylum (*Bacteroidia, Bacteroidales, Muribaculaceae, Bacteroidaceae, Bacteroides, Prevotella*) and *Proteobacteria* phylum (*Alphaproteobacteria, Rhodospirillales*) had significant differences in AM group rats compared to HF group rats (Fig. 2c). Simultaneously, LEfSe analysis elucidated the genus level differences such that HF group rats was more abundant in species of *Christensenellaceae* compared with SV group rats, whereas there was only one genus (*Paenalcaligenes*) had significant differences in SV group rats compared with HF group rats (Fig. 2d).

### CBPs changes serum BAs pool, which is related in gut microbial composition

**Fig. 3:**
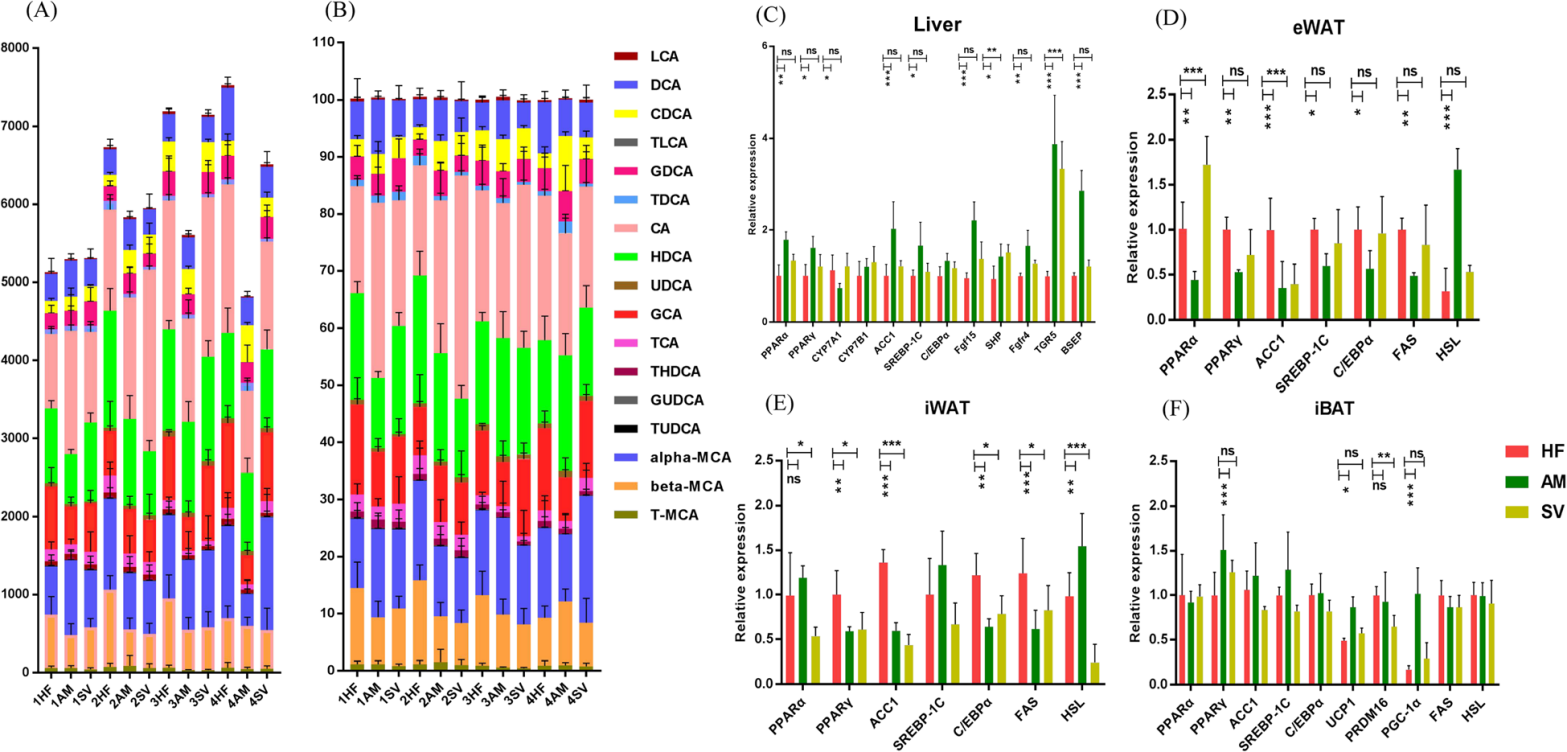
CBPs changes serum BAs pool and composition and regulating the mRNA expression of genes involved in lipid metabolism, energy homeostasis and thermogenesis. **a** Serum BAs pool absolute contents and **b** Serum BAs pool relative contents in HF, AM and SV group rat at day 10, day 20, day 30, day 40 after diet intervention. **c-f** The mRNA expression of genes in liver, epididymal adipose tissue (eWAT), inguinal adipose tissue (iWAT) and interscapular brown adipose tissue (iBAT) were determined by RT- PCR analysis. and relative gene pressions were normalized with β-actin. Values are presented as mean ± SEM. *P < 0.05; **P < 0.01; ***P < 0.001; ns *P* > 0.05

BAs synthesis is an important pathway for catabolism of cholesterol and is closely regulated by complex mechanisms that are not completely understood. BAs were considered as mediators of metabolism, alteration the BAs homeostasis will cause many diseases such as obesity, diabetes, nonalcoholic fatty liver disease and hyperlipemia [26]. We anticipated that CBPs treatment could shift the BAs pool in HFD-fed rats. As can be seen from Fig. 3a, the total serum BAs content of AM group rats increased 20 days ago and then decreased gradually after 20 days. Nevertheless, within 40 days of high-fat diet feeding, the total serum BAs content increased continuously in HF group rats. These results suggested that CBPs can significantly improve the shift of BAs pool which induced by HFD in obese rats. The total serum BAs content of SV group rats increased continuously and decreased slightly after 30 days. Furthermore, the relative content of cholic acid (CA), deoxycholic acid (DCA) and taurohyodeoxycholic acid (THDCA) were decreased gradually in AM group rats, while the relative content of chenodeoxycholic acid (CDCA), hyodeoxycholic acid (HDCA), ursodeoxycholic acid (UDCA) and β-muricholic acid (β-MCA) were enhanced in AM group rats (Supplementary Fig. 3). Compared with AM group rats, the relative content of CA and DCA was higher and relative content of β-MCA and HDCA were lower in HF group rats after 40 days. In addition, the relative content of UDCA increased slightly and the relative content of TUDCA and THDCA decreased in SV group rats within 40 days.

Correlation coefficients between the relative content of serum BAs and the relative abundance of gut bacteria at genus-level were shown in Table 4. Several BAs correlated with specific bacterial genera. *Bacteroides* was positively correlated with CDCA, HDCA and negatively correlated with DCA, GCA. Similarly, *Prevotella* was positively correlated with CDCA, HDCA, β-MCA and negatively correlated with DCA, GCA, TUDCA. Interestingly, *Acetitomaculum* and *Prevotella* have the same trend of association with BAs. In addition, β-MCA positively correlated with *Akkermansia*. On the contrary, *Desulfovibrio* was negatively correlated with β-MCA and CDCA.

### CBPs regulates gene expression in liver and adipose tissues of HFD-fed rats

To further explore the molecular mechanism of CBPs improving obesity in HFD-fed rats, we evaluated the gene expression of lipogenesis, lipolysis and BAs metabolism in liver and adipose tissues. In the liver tissue, compared with HF group rats, AM group rats significantly enhanced the mRNA expression of peroxisome proliferator-activated receptor α (PPARα), peroxisome proliferator-activated receptor γ (PPARγ), small heterodimer partner (SHP), G protein-coupled bile acid receptor (TGR5), fibroblast growth factor 15 (FGF15), fibroblast growth factor 4 (Fgfr4), bile salt export protein (BSEP) and downregulated the mRNA expression of cholesterol-7a-hydroxylase (CYP7A1) (Fig. 3c). The results indicated that CBPs treatment could alleviate the disorder of hepatic BAs metabolism and fat accumulation. Simvastatin treatment also partially improved hepatic BAs metabolism by up-regulating SHP and TGR5 gene expression (Fig. 3c).

In the eWAT, CBPs treatment markedly downregulated the mRNA expression of PPARα, PPARγ, acetyl-coenzyme A carboxylase 1 (ACC1), sterol regulatory element binding protein-1c (SREBP-1c), CCAAT enhancer binding protein α (C/EBPα), fatty acid synthetase (FAS) and enhanced the mRNA expression of hormone-sensitive lipase (HSL) in AM group rats compared with HF group rats (Fig. 3d). In accordance with the eWAT, PPARγ, ACC1, C/EBPα, FAS were dramatically downregulated and HSL was upregulated in the iWAT after CBPs treatment (Fig. 3e). Simvastatin treatment reduced mRNA abundance of ACC1 and increased mRNA abundance of PPARα in the eWAT compared with HF group rats (Fig. 3d). Meanwhile, the mRNA expression of PPARα, PPARγ, ACC1, C/EBPα and FAS were downregulated slightly to improve fat accumulation in the iWAT after simvastatin treatment (Fig. 3e). In the iBAT, compared with HF group rats, CBPs treatment positively regulated the mRNA expression of peroxisome proliferator-activated receptor γ co-activator 1α (PGC-1α), PPARγ and upregulation of uncoupling protein 1 (UCP1) in AM group rats, while simvastatin treatment had no similar effect in SV group rats (Fig. 3f). Consequently, we could conclude that CBPs can improve lipid metabolic syndrome in HFD-fed rats by regulating the related mRNA expression of lipogenesis and lipolysis in the WAT and modulate energy homeostasis and thermogenesis in the iBAT.

### Fecal microbiota transplantation (FMT) from CBPs-treated rats remodels gut microbiota and improves dyslipidemia in HFD-fed rats

**Fig. 4:**
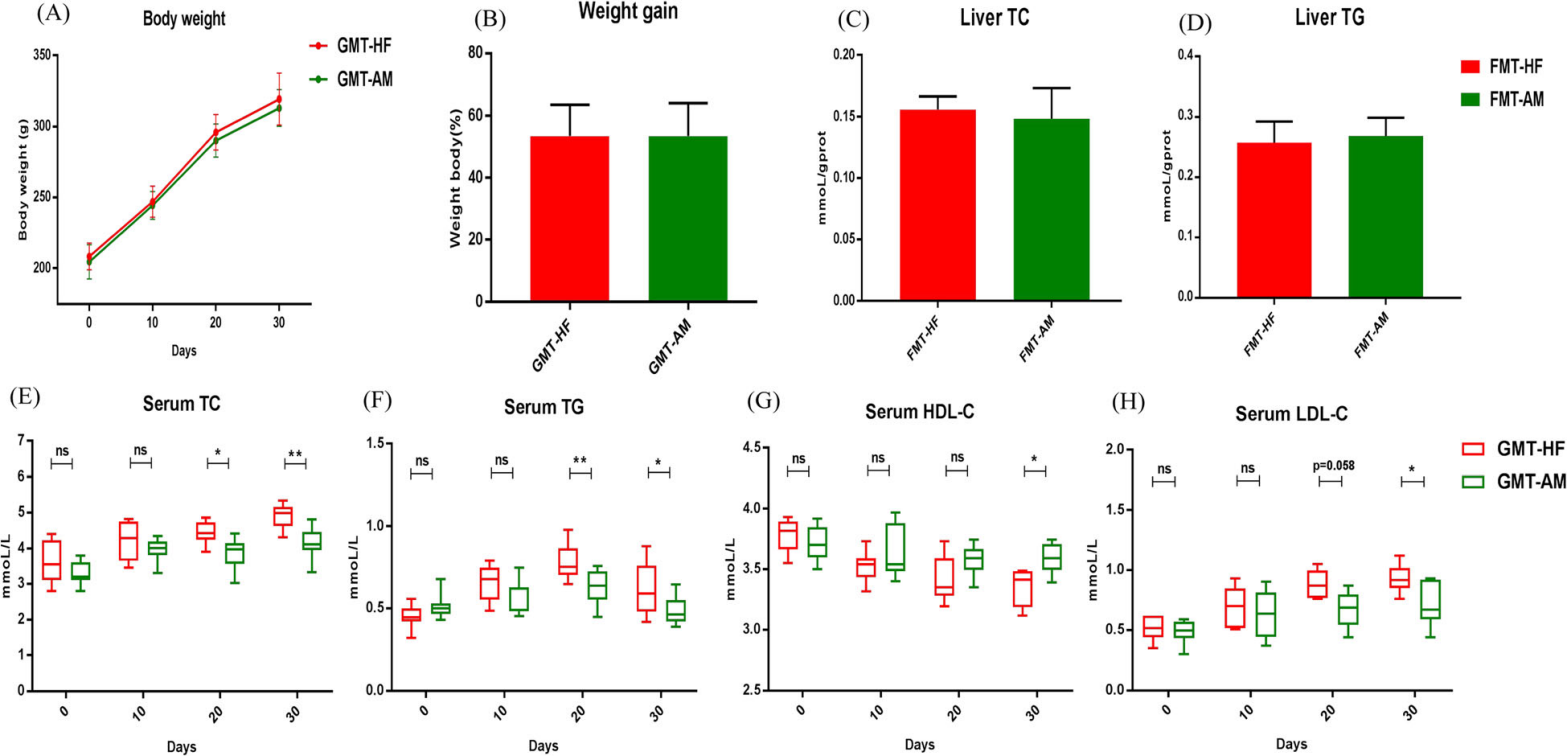
Although fecal microbiota transplantation from CBPs treatment rat failed to reduce body weight, it could improve dyslipidemia in HFD-induced rat. **a** Body weight (g), **b** Weight gain, **c-f** Serum concentrations of TC, TG, HDL-C and LDL-C (mmol/L). Values are means ± SEMs. *P < 0.05, **p < 0.01 and ***p < 0.001

We investigated the FMT from CBPs-treated rats remodeled gut microbiota and improved lipid metabolism in HFD-fed rats. As shown in Fig. 4 A-B, there were no significant difference in body weight and weight gain between the FMT-HF and FMT-AM group rats within 30 days. And the weight of liver, kidney, spleen, iWAT, eWAT, pWAT and iBAT were no significant difference between the FMT-HF and FMT-AM group rats (Supplementary Fig. 5). However, FMT from CBPs-treated rats could significantly reduce serum TC, TG, LDL-C and increase HDL-C in FMT-AM group rats compared with FMT-HF group rats (Fig. 4 E-H). In liver, the concentration of TC and TG showed no significant difference in the two groups rats (Fig. 4 C-D).

**Fig. 5:**
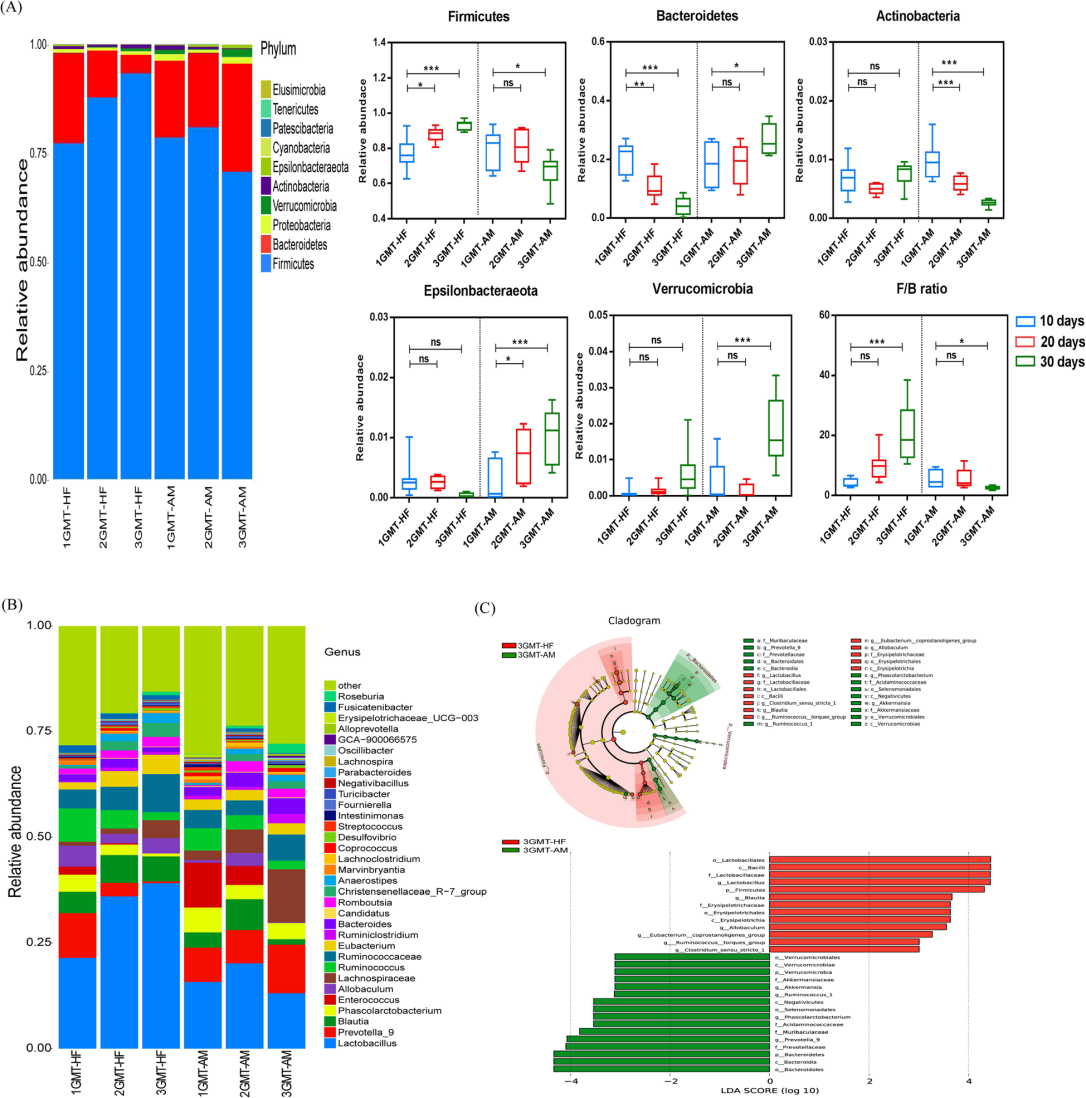
Fecal microbiota transplantation from CBPs treatment rat improved gut microbiota in HFD-induced rat. **a** Microbiota compositions at the phylum level, **b** Microbiota compositions at the genus level in FMT-HF and FMT-AM group at day 10, day 20, day 30 after fecal microbiota transplantation from CBPs treatment rat. The linear discriminant analysis (LDA) effect size (LEfSe) was used to identify the biomarkers with significant differences between the two groups: **c** FMT-HF vs FMT-AM. Values are presented as mean ± SEM. *P < 0.05; **P < 0.01; ***P < 0.001; ns *P* > 0.05

Furthermore, to reveal the effects of FMT on the gut microbial structure, we sequenced the fecal bacterial 16S rRNA after 10, 20 and 30 days in FMT-HF group rats and FMT-AM group rats. The ACE and Chao1 indexs were increased gradually, while the shannon and simpson indexes did not change significantly within 30 days in FMT-AM and FMT-HF group rats (Table 3). At the phylum level, FMT from CBPs-treated rats tended to increase the relative abundance of *Bacteroidetes*, *Verrucomicrobia* and *Epsilonbacteraeota* but decrease the relative abundance of *Firmicutes* and *Actinobacteria* within 30 days. Conversely, the relative abundance of *Firmicutes*, *Actinobacteria* were higher and *Bacteroidetes*, *Verrucomicrobia* and *Epsilonbacteraeota* were lower in FMT-HF group rats compared with FMT-AM group rats (Fig. 5a). The F/B ratio was increased dramatically in FMT-HF group rats, whereas FMT from CBPs treatment rats reversed this trend significantly after 30 days.

**Table 3:** The ACE, Chao1, Shannon and Simpsom index in FMT-HF and FMT-AM group rat at day 10, day 20, day 30 after fecal microbiota transplantation from CBPs treatment rat

Sample	FMT-HF			FMT-AM		
	1FMT-HF	2FMT-HF	3FMT-HF	1FMT-AM	2FMT-AM	3FMT-AM
ACE	301.38 ± 20.60^b^	325.24 ± 23.25^a^	339.13 ± 26.20^a^	257.44 ± 55.52^c^	311.19 ± 47.76^b^	357.40 ± 30.53^a^
Chao1	304.81 ± 26.05^b^	329.00 ± 26.91^a^	343.19 ± 23.43^a^	261.16 ± 56.58^b^	322.07 ± 48.93^a^	358.92 ± 32.51^a^
Shannon index	5.03 ± 0.19	5.34 ± 0.33	5.15 ± 0.40	5.07 ± 0.38	5.27 ± 0.42	5.68 ± 0.26
Simpsom index	0.94 ± 0.01	0.92 ± 0.03	0.92 ± 0.03	0.92 ± 0.02	0.93 ± 0.04	0.95 ± 0.02

At the genus level, the relative abundance of *Bacteroides, Prevotella* and *Akkermansia* was higher, while the relative levels of *Blautia* and *Streptococcus* were markedly lower in FMT-AM group rats compared with FMT-HF group rats (Supplementary Fig. 6). In FMT-HF group rats, the relative abundance of *Prevotella*, *Phascolarctobacterium* was reduced and the relative abundance of *Lactobacillus*, *Eubacterium* was increased gradually. The LEfSe analysis results indicated the relative abundance of *Firmicutes* phylum (*Bacilli, Lactobacillales, Lactobacillaceae, Lactobacillus, Erysipelotrichia, Erysipelotrichales, Erysipelotrichaceae, Allobaculum, Blautia, Eubacterium, Ruminococcus, Clostridium*) in FMT-HF group rats was significantly increased compared with FMT-AM group rats (Fig. 5c). The *Bacteroidetes* phylum (*Bacteroidia, Bacteroidales, Bacteroidaceae, Prevotellaceae, Prevotella, Muribaculaceae*), *Verrucomicrobia* phylum (*Verrucomicrobiae, Verrucomicrobiales, Akkermansiaceae, Akkermansia*) and *Firmicutes* phylum (*Negativicutes, Selenomonadales, Acidaminococcaceae, Phascolarctobacterium*) were identified by LEfSe as discriminative taxa in FMT-AM group rats compared with FMT-HF group rats (Fig. 5c).

## Discussion

This is the first report of CBPs treatment influencing host gut microbiota and lipid metabolism in HFD-fed rats. We presented evidence that CBPs treatment effectively prevent obesity and alleviate lipid metabolic syndrome in HFD-fed rats. Moreover, altered BAs profile may affect the brown fat activation by regulating energy homeostasis and thermogenesis in the host. Some previous reports have shown that HFD treatment resulted in reduced intestinal microbial richness and diversity [13, 27]. Our results did not show alterative gut microbial diversity in HF, AM and SV group rats. Interestingly, the intestinal microbial richness in HF group increased after 10 days with HFD treatment, whereas the intestinal microbial richness did not change in AM and SV group rats. To sum up, intestinal microbial richness and diversity have no connection with the development of obesity in our study.

In addition, the increased F/B ratio has been associated with obesity and increased energy harvest by the gut microbiota [13]. Our results also show a marked reduction of F/B ratio in AM group rats, which was significantly different compared with HF group rats after 40 days (*P*<0.001). *Akkermansia*, as a new functional microbe, belongs to the *Verrucomicrobia* phylum. A great deal of evidence has proved that *Akkermansia* plays a critical role in metabolic homeostasis and reduce weight gain and metabolic syndrome in the host [28, 29]. *Clostridium* and *Lachnospiraceae* were enriched in mice fed with HFD diet [29, 30]. *Romboutsia* was negatively associated with the body weight, fasting serum glucose and insulin [31]. *Bacteroides* and *Prevotella* also showed beneficial effects for weight loss [32]. Dietary interventions and nutritional modulation can reduce opportunistic pathogens *Desulfovibrio* [33]. In our study, the results demonstrated HFD treatment increased *Firmicutes* and its genus *Romboutsia*, *Clostridium*, *Lachnospiraceae_NK4A136_group* and decreased *Bacteroidetes* and its genus *Bacteroides* and *Prevotella*. However, CBPs can significantly change these trends. *Akkermansia*, *Bacteroides* and *Prevotella* were significantly enriched and *Desulfovibrio*, *Lachnoclostridium* and *Lachnospiraceae_NK4A136_group* were depleted with the extension of CBPs treatment time in AM group rats. Therefore, CBPs treatment prevent HFD-induced obesity and complications by modulating the gut microbial composition in multiple ways. A cladogram generated from the LEfSe analysis indicated the most differentially abundant taxa enriched in the gut microbiota of AM group rats and HF group rats. The results further illustrated *Firmicutes* (*Romboutsia, Ruminococcaceae, Turicibacter, UBA1819, Anaerotruncus*) positively correlated with weight gain in HFD-fed rats, whereas CBPs treatment observably enriched *Bacteroides*, *Prevotella* and *Akkermansia* in HFD-fed rats (Fig. 2c). Therefore, our results indicated that the *Bacteroides*, *Prevotella* and *Akkermansia* can be used as biomarkers for evaluating alleviation of obesity. After 40 days, the LEfSe analysis demonstrated there was no significant difference in the above well-known beneficial bacteria and opportunistic pathogens bacteria between SV group rats and HF group rats, which indicated that simvastatin failed to alter gut microbial composition in HFD-fed rats (Fig. 2d).

We believed that CBPs treatment can markedly improve BAs metabolism though altering gut microbial components in HFD-fed rats. BAs as important signaling molecules regulate host metabolism through activation two major BAs receptors: farnesoid X receptor (FXR) and TGR5 [34]. The FXR, as an important nuclear receptor of BAs, plays a critical role for BAs metabolism. FXR negative feedback regulates BAs synthesis through at least two distinct mechanisms:1) Activated FXR upregulates the expression of transcription SHP and then downregulates the expression of CYP7A1 by induction of SHP activity, thus inhibiting the conversion of cholesterol to BAs in liver. 2) After ileal FXR is triggered by BAs, which induces production of FGF15. FGF15 acts on hepatocytes through activation FGFR4 to repress transcription of CYP7A1 [35, 36]. In addition, activated FXR induces the expression of the transporters BSEP that secrete bile salts from hepatocytes into the canaliculi [35]. It is known that CDCA is the most efficacious ligand of FXR [34]. After CBPs treatment, the relative content of CDCA dramatically increased within 40 days, which was positively correlated with *Bacteroides* and *Prevotella.* We believed that increased CDCA levels effectively activated FXR signaling pathways and thus inhibit BAs synthesis in liver. Besides, we found that HFD treatment enhanced the relative content of CA and DCA in obese rats, whereas CBPs treatment significantly decreased the relative content of CA. Previous studies have shown that CA-containing diet supplement resulted in increased F/B ratio, which was also seen in obese mice [37]. Our results also indicated that CA was closely associated with obesity in HFD-fed rats. DCA is produced through 7a-dehydroxylation of primary BAs (CA and CDCA) with the participation of gut microbiota such as *Eubacterium* and *Clostridium* [38]. The high level of DCA has been demonstrated to induce adverse effects on health [25]. We found that relative content of DCA increased in HF group rats. Nevertheless, after CBPs treatment, the relative content of DCA decreased significantly within 40 days. As can be seen from Table 4, DCA was positively correlated with *Clostridium*, *Eubacterium*, *Ruminococcaceae* and negatively correlated with *Bacteroides, Prevotella* in AM group rats. *Ruminococcaceae* are thought to produce 7a-dehydroxylase, which increases DCA level in feces of cirrhosis patients [39]. Hence, our findings further confirmed that there was a close relationship between BAs metabolism and gut microbial composition in HFD-fed rats.

**Table 4:**
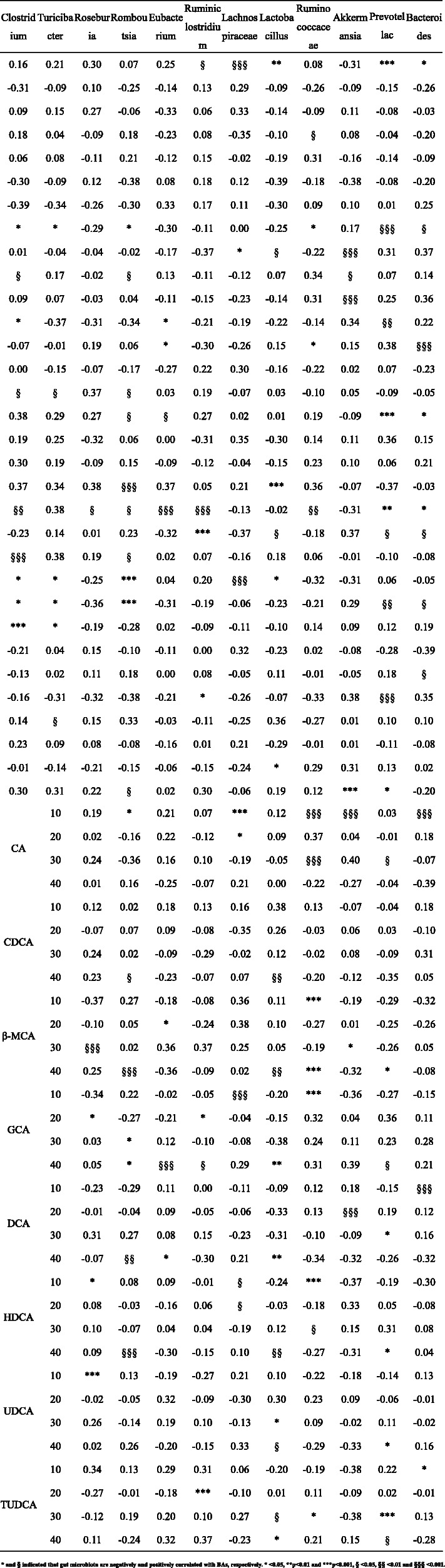
Correlation between key gut microbiotal and BAs in CBPs-treated rat

This study also revealed that dietary supplementation of CBPs regulates the mRNA expression related to lipogenesis, lipolysis, energy homeostasis and thermogenesis in liver and adipose tissues, which was most likely mediated through FXR and TGR-5 signaling pathway to improve lipid metabolism. In liver, after CBPs treatment, the mRNA expression of SHP, FGF15, FGFR4 and BSEP were upregulated and CYP7A1 was downregulated in HFD-fed rats. Therefore, we thought that CBPs reduces total serum BAs by inhibiting BAs synthesis in liver and promoting BAs secretion into the canaliculi. Moreover, FXR also participates in hepatic lipid homeostasis. SREBP1-c, a well-known critical transcription factor, regulates expression of the downstream marker molecules such as FAS, ACC1, HSL to result in the enhancement of fatty acid synthesis and accumulation of TG [40]. The expression of SREBP-1c was repressed by activation FXR through FXR/SHP pathway, which inhibited hepatic lipogenesis by regulation cascade reaction of lipid synthesis [41, 42]. Simultaneously, FXR promotes free fatty acids (FFA) β oxidation by activation the expression of PPARα, a regulator of triglyceride metabolism [43]. Surprisingly, compare with HF group rats, PPARγ, ACC1 and SREBP-1c, as regulators of lipid synthesis, were upregulated in AM group rats. These results were similar to the previous reports that melatonin positively regulated mRNA expression of PPARγ and ACC1 in HFD-fed mice [44]. The specific reasons need further exploration. Therefore, we speculated that CBPs improves hepatic lipid metabolism in HFD-fed rats through FXR/PPARα axis pathway rather than FXR/SHP/SREBP-1c axis. In addition, TGR5 activation by BAs could inhibit fat accumulation in the liver [26, 45]. We found that CBPs and simvastatin treatment dramatically upregulated the mRNA expression of TGR5 in HFD-fed rats. However, simvastatin treatment failed to regulate the mRNA expression of SREBP, FGF15, FGFR4 and CYP7A1 by activating FXR. Although the effects of altered BAs profile on related genes expression of lipogenesis, lipolysis and BAs metabolism need further investigation, the striking finding from our study was that altered BAs profile found in AM group rats likely contributes to activate FXR and TGR5 in the liver and then improves hepatic fat accumulation and BAs metabolism. Simvastatin, as HMG-CoA reductase inhibitors, also slightly improves hepatic lipid metabolism via activated TGR5 pathway rather than via the activated FXR pathway.

PPARγ,SREBP-1c and C/EBPα are a series of transcription factors that regulate lipogenesis and lipolysis by controlling the expression of several enzymes in WAT such as ACC1, FAS, HSL and so on. After CBPs treatment, the mRNA expression of PPARγ, SREBP-1c and C/EBPα were downregulated in AM group rats compared with HF group rats in iWAT. Accordingly, the mRNA expression of lipid synthesis rate-limiting enzyme ACC1 and FAS were downregulated and lipidolysis rate-limiting enzyme HSL was upregulated in AM group rats. These results indicated that CBPs treatment can significantly inhibit lipogenesis and promote lipolysis in WAT of HFD-fed rats. Similar results were also found in eWAT. Simvastatin treatment may suppress lipogenesis in HFD-fed rats. However, it can not promote lipolysis in WAT, which even inhibited the mRNA expression of HSL in iWAT.

BAT (brown adipose tissue) is the main site of thermogenesis in mammals, which was also found to oxidize fatty acids without ATP production contributes to energy expenditure [46]. Activated BAT could effectively prevent obesity and related metabolic diseases [47]. In our study, the mRNA expression of PPARγ, UCP1 and PGC-1α were upregulated markedly in iBAT of AM group rats. PGC-1α as a transcription-assisted activator regulates the expression of PPARγ, including the induction of UCP1 gene expression. UCP1 has classically been regarded as a marker of BAT, which maintains energy expenditure and thermogenesis in the host [48]. Besides, BAs are thought to enhance HFD-induced thermogenesis through upregulation UCP1 in BAT, which may be related to the activation of TGR5 [46]. Activated UCP1 can increase the energy expenditure and thermogenesis in the host, thus reducing the body weight. We confirmed that CBPs treatment could reduce the body weight of HFD-fed rats by accelerating energy homeostasis and thermogenesis in iBAT, but simvastatin had no such effect.

FMT experiments revealed that FMT from HF group rats can accelerate dyslipidemia in HFD-fed rats. The symptoms were alleviated by treatment with FMT from CBPs-treated rats (Fig. 4e-h), indicating that gut microbiota participates in lipid metabolism in HFD-fed rats. Simultaneously, we found that the fecal resuspensions from AM and HF group rats could be stably colonized in FMT-AM and FMT-HF group rats, which reshaped gut microbiota of HFD-fed rats. Similar to AM group rats, the relative abundance of *Firmicutes* and F/B ratio declined and the relative abundance of *Bacteroidetes* increased in FMT-AM group rats after 30 days, whereas the relative abundance of *Firmicutes, Bacteroidetes* and F/B ratio in FMT-HF group rats showed a reverse trend compared with FMT-AM group rats (Fig. 5a). Furthermore, the relative abundance of *Bacteroides*, *Prevotella* and *Akkermansia* increased in FMT-AM group rats (Supplementary Fig. 6). Therefore, we considered *Bacteroides*, *Prevotella* and *Akkermansia* may be critical contributors for improving lipid metabolism in HFD-fed rats. Intriguingly, the relative abundance of *Lactobacillus* enhanced remarkably in FMT-HF group rats, which is in contrast with the previous studies that *Lactobacillus* prevented HFD-induced obesity and hepatic steatosis [49, 50]. Nevertheless, our results supported another standpoint that there was a positive correlation between *Lactobacillus* and obesity [51, 52].

## Conclusions

Much evidence exists indicating that berries rich in polyphenols have a variety of physiological and pharmacological activities. Our research indicated that CBPs treatment altered gut microbial composition and improved lipid metabolism with the extended treatment time in HFD-fed rats. The mRNA expression related to BAs metabolism, lipogenesis and lipolysis in liver and adipose tissues were closely related to gut microbiota components. Moreover, altered gut microbiota components may affect the brown fat activation by regulating energy homeostasis and thermogenesis through modulated the BAs metabolism in the host. In addition, our findings opened the possibility that FMT from healthy rats reshaped gut microbiota and improved dyslipidemia in HFD-fed rats, which is a powerful evidence for the treatment of obesity by FMT. Consequently, CBPs treatment poses potential as an effective therapeutic measure to restore gut microbiota homeostasis and metabolic disturbances associated with obesity and related chronic disease.

## Supplementary information

**Additional file 1 Table S1**. Chromatography condition. **Table S2**. Primer sequences. **Figure S1**. The weight (g) of heart, kidney, spleen, lung, pancreas and testicle in the Control, HF, AM and SV group rat. Values are means ± SEMs. **Figure S2.** Gut microbiota compositions at the genus level in HF, AM and SV group at day 10, day 20, day 30, day 40 after diet intervention. Values are presented as mean ± SEM. *P < 0.05; **P < 0.01; ***P < 0.001; ns *P* > 0.05. **Figure S3.** The relative contents of bile acids in HF, AM and SV group rat at day 10, day 20, day 30, day 40 after diet intervention. Values are presented as mean ± SEM. *P<0.05; **P<0.01; ***P<0.001; ns P>0.05. **Figure S4**. Microbiota compositions at the genus level in HF, AM and SV group at day 40 after diet intervention. Values are presented as mean ± SEM. *P<0.05; **P<0.01; ***P<0.001; ns P>0.05. **Figure S5**. The weight (g) of liver, kidney, spleen, iWAT, eWAT, pWAT and iBAT in FMT-HF and FMT-AM group rat. Values are means ± SEMs. **Figure S6.** Microbiota compositions at the genus level in FMT-HF and FMT-AM group at day 10, day 20, day 30 after microbiota transplantation from CBPs treatment rat. Values are presented as mean ± SEM. *P < 0.05; **P < 0.01; ***P < 0.001; ns P > 0.05.

## Data Availability

All raw sequences from 16S rRNA gene-based analysis have been uploaded to GigaDB.

## Abbreviations

CBPs: Chokeberry polyphenol
HFD: High fat diet
F/B ratio: *Firmicutes*/*Bacteroidetes* ratio
FMT: Fecal microbiota transplantation
WAT: White adipose tissue
eWAT: Epididymal adipose tissue
iWAT: Inguinal adipose tissue
pWAT: Perirenal adipose tissue
iBAT: Interscapular brown adipose tissue
BAs: Bile acids
