# Supplementary material for: The polyphenol-rich extract from chokeberry (Aronia melanocarpa L.) modulates gut microbiota and improves lipid metabolism in diet-induced obese rats
Source: Nutr Metab (Lond). 2020 Jul 7;17:54. doi: 10.1186/s12986-020-00473-9 (PMC7339576; doi:10.1186/s12986-020-00473-9)
Supplement: Supplementary file 1 — Additional file 1 Table S1. Chromatography condition. Table S2. Primer sequences. Figure S1. The weight (g) of heart, kidney, spleen, lung, pancreas and testicle in the Control, HF, AM and SV group rat. Values are means ± SEMs. Figure S2. Gut microbiota compositions at the genus level in HF, AM and SV group at day 10, day 20, day 30, day 40 after diet intervention. Values are presented as mean ± SEM. *P < 0.05; **P < 0.01; ***P < 0.001; ns P > 0.05. Figure S3. The relative contents of bile acids in HF, AM and SV group rat at day 10, day 20, day 30, day 40 after diet intervention. Values are presented as mean ± SEM. *P<0.05; **P<0.01; ***P<0.001; ns P>0.05. Figure S4. Microbiota compositions at the genus level in HF, AM and SV group at day 40 after diet intervention. Values are presented as mean ± SEM. *P<0.05; **P<0.01; ***P<0.001; ns P>0.05. Figure S5. The weight (g) of liver, kidney, spleen, iWAT, eWAT, pWAT and iBAT in FMT-HF and FMT-AM group rat. Values are means ± SEMs. Figure S6. Microbiota compositions at the genus level in FMT-HF and FMT-AM group at day 10, day 20, day 30 after microbiota transplantation from CBPs treatment rat. Values are presented as mean ± SEM. *P < 0.05; **P < 0.01; ***P < 0.001; ns P > 0.05. [file 12986_2020_473_MOESM1_ESM.zip › Supplyment Table and Figure.doc]

**Supplyment Table 1 Chromatography condition**

| Time (min) | A% (Methanol) | B% (2mmol/L Ammonium acetate) |
| --- | --- | --- |
| 0 | 30 | 70 |
| 1.5 | 50 | 50 |
| 5.0 | 60 | 40 |
| 10.0 | 78 | 22 |
| 15.0 | 95 | 5 |
| 15.1 | 30 | 70 |
| 19.0 | 30 | 70 |

**Supplyment Table 2 Primer sequences**

| Gene Name | Size（bp） | Forward | Reverse |
| --- | --- | --- | --- |
| Actin | 111 | cgttgacatccgtaaagacc | ctaggagccagggcagtaatc |
| PPARα | 122 | tcacacaatgcaatccgttt | gccagagatttgaggtctgc |
| PPARγ | 108 | gaccactcccattcctttga | aaccattgggtcagctcttg |
| CYP7A1 | 99 | gagaacgggttgattccgta | aaaaacgtgaccatgcttcc |
| CYP7B1 | 127 | tcatccgtgaagtgcaagag | ggagcatcgaagacttctgg |
| ACC1 | 97 | tgaggaggaccgcatttatc | gcatggaatggcagtaaggt |
| SREBP-1C | 115 | ccagcctttgaggataacca | tgcaggtcagacacaggaag |
| C/EBPα | 125 | gccaagaagtcggtggataa | aacaccttctgctgcgtctc |
| Fgf15 | 114 | aaagtggagtgggcgtattg | agtggaccttcatccgacac |
| SHP | 108 | ctctcttcctgcttgggttg | caccactgttgggttcctct |
| Fgfr4 | 109 | tatcaccgacaagcaatcca | cagaggacctcgactccaag |
| BSEP | 113 | ccttctcgacccgatattca | agctgaatgctggtgctttt |
| UCP1 | 100 | aatcagctttgcttccctca | cgagatcttgcttcccaaag |
| HSL | 92 | gagacggaggaccattttga | cggaggtctctgaggaacag |
| FAS | 92 | aagatcccggaaagcaagat | tgataccagcactggagcag |
| PGC-1α | 117 | gatgctcccaattccagtgt | ctctgcactggcaaagtcaa |

**Supplementary Fig.1** **The weight (g) of heart, kidney, spleen, lung, pancreas and testicle in the Control, HF, AM and SV group rat. Values are means ± SEMs.**

**Supplementary Fig.2 Gut microbiota compositions at the genus level in HF, AM and SV group at day 10, day 20, day 30, day 40 after diet intervention. Values are presented as mean ± SEM. *P<0.05; **P<0.01; ***P<0.001; ns P>0.05.**

**Supplementary Fig.3 The relative contents of bile acids in HF, AM and SV group rat at day 10, day 20, day 30, day 40 after diet intervention. Values are presented as mean ± SEM. *P<0.05; **P<0.01; ***P<0.001; ns P>0.05.**

**Supplementary Fig.4 Microbiota compositions at the genus level in HF, AM and SV group at day 40 after diet intervention. Values are presented as mean ± SEM. *P<0.05; **P<0.01; ***P<0.001; ns P>0.05.**

**Supplementary Fig.5** **The weight (g) of liver, kidney, spleen, iWAT, eWAT, pWAT and iBAT in FMT-HF and FMT-AM group rat. Values are means ± SEMs.**

**Supplementary Fig. 6 Microbiota compositions at the genus level in FMT-HF and FMT-AM group at day 10, day 20, day 30 after microbiota transplantation from CBPs treatment rat. Values are presented as mean ± SEM. *P<0.05; **P<0.01; ***P<0.001; ns P>0.05.**
